# Recent Developments in Photofunctional Nanomaterials and Nanostructures for Emitting, Manipulating, and Harvesting Light

**DOI:** 10.3390/nano14242023

**Published:** 2024-12-16

**Authors:** Zhixing Gan

**Affiliations:** Center for Future Optoelectronic Functional Materials, School of Computer and Electronic Information/School of Artificial Intelligence, Nanjing Normal University, Nanjing 210023, China; zxgan@njnu.edu.cn

## 1. Introduction

Photofunctional nanomaterials and nanostructures that can emit, manipulate, convert, and utilize photons in diverse forms have profound meanings, from fundamental understandings to applications. Thus, photofunctional nanomaterials and nanostructures have stimulated trans-disciplinary interests in the fields of physics, chemistry, material science, biology, photons, and engineering while also stimulating scientific breakthroughs in the fields of photovoltaics, photolithography, photoelectronics, photocatalysis, photobiology and phototherapy, photosynthesis, and optical sensing. Recently, photofunctional materials have been developed for storing and processing optical information [1,2,3]. Neuromorphic optoelectronic devices integrating sensing, storage, and computing abilities have been demonstrated [4,5,6]. Photofunctional nanomaterials and nanostructures, with their unique appeal, are attracting a growing number of researchers to advance the development of this field.

## 2. An Overview of Published Articles

This Special Issue brings together eleven articles, including one review article and ten research articles. Four of these articles focus on development of photofunctional metastructures, such as metamirrors, nanotube photonic crystasl, topological photonic devices, and metasurface filters.

Li et al. designed the chiral metamirrors with circular dichroisms of about 0.4 in visible reflection [7]. The chiral metamirrors show high reflectance for right-handed circular polarization with preserved handedness and strongly absorbed left-handed circular polarization on chiroptical resonant wavelengths.

Meng et al. developed a novel bi-layer structure consisting of a top nanotube layer and a bottom nanotube photonic crystal layer in which the photonic bandgap of bottom TiO_2_ nanotube photonic crystals can be precisely adjusted by modulating the anodization parameters [8]. The overlapping between the photonic bandgap of photonic crystals with the electronic bandgap of TiO_2_ leads to the boosted ultraviolet light absorption of the top TiO_2_ layer and enhanced photon-to-current conversion efficiency. This research offers an effective strategy for improving the performance of photoelectrochemical water splitting through intensifying light–matter interactions.

Su et al. computationally proposed a photonic device for the 1550 nm communication band in which topologically protected electromagnetic modes of a high quality can be selectively triggered and modulated on demand [9]. The topological photonic devices can realize Fano lines on the spectrum and show high-quality localized modes by tuning the coupling strength between the zero-dimensional valley corner states and the one-dimensional valley edge states, providing a promising approach for multi-dimensional optical field manipulation in integrated nanophotonic devices.

Wang et al. proposed a compact snapshot compressive spectral-imaging (SCSI) system that leverges the spectral modulations of metasurfaces with dual-channel switchable metasurface filters and employs a deep-learning-based reconstruction algorithm [10]. The proposed SCSI system integrates dual-channel switchable metasurface filters using twisted nematic liquid crystals and anisotropic titanium dioxide nanostructures. The proposed hyperspectral-imaging technology demonstrates superior reconstruction quality and speed compared to those of the traditional compressive hyperspectral image recovery methods. This device is expected to be applied in various areas, such as object detection, face recognition, food safety, biomedical imaging, agriculture surveillance, etc.

The other six research articles focused on the development of photofunctional nanomaterials, with three articles concentrating on light-emitting materials and the final three articles examining light-harvesting materials.

Tselekidou et al. investigated the optical and photophysical characteristics of blue-emitting polymers to promote the understanding of the fundamental mechanisms of color purity and its stability during the operation of Organic Light-Emitting Diode (OLED) devices [11].

Zhang et al. hypothesized that the blue emission of carbon dots (CDs) could be ascribed to the surface states induced by the C–O and C=O groups, while the green luminescence may originate from the deep energy levels associated with the O–C=O groups according to microstructure characterizations, optical measurements, and ultrafiltration experiments [12].

Tardío et al. synthesized and crystallized a set of novel Donor–Acceptor–Donor (D-A-D) benzoselenadiazole derivatives in nanocrystals [13]. The correlation between their chemical structures and the waveguided luminescent properties were explored. The findings revealed that all crystals exhibited luminescence and active optical waveguiding, demonstrating their ability to adjust their luminescence within a broad spectral range of 550–700 nm through suitable chemical functionalization.

Goponenko et al. proposed a novel hydrophobic coating based on a polydimethylsiloxane layer with embedded red-emitting Y_2_O_3_:Eu^3+^ particles as UV radiation screening and conversion layers for solar cells, resulting in a notable increase in power conversion efficiency by ~9.23% [14]. The developed coating can endure tough environmental conditions, making it potentially useful as a UV-protective, water-repellent, and efficiency-enhancing coating for solar cells.

Slimani et al. studied the intense-pulsed-light-induced crystallization of SnO_2_ thin-films using only 500 μs of exposure time [15]. They demonstrated that light-induced crystallization yields improved topography and excellent electrical properties through enhanced charge transfer, improved interfacial morphology, and better ohmic contact compared to thermally annealed SnO_2_ films, showing great potential for improved perovskite solar cell manufacturability.

Chaperman synthesized self-doped CuS nanoparticles via a microwave-assisted polyol process to act as co-catalysts to TiO_2_ nanofiber-based photoanodes for visible light-assisted water electrolysis [16]. These low-cost and easy-to-achieve composite materials allow for an improved overall efficiency of water oxidation (and consequently hydrogen generation at the Pt counter electrode) in passive electrolytes, even with a 0 V bias.

Furthermore, in the mini-review, Guo et al. summarized recent research progress on the Rashba effect of two-dimensional (2D) organic–inorganic hybrid perovskites [17]. The origin and magnitude of Rashba spin splitting, the layer-dependent Rashba band splitting of 2D perovskites, and the Rashba effect of different 2D perovskites are discussed. Moreover, related applications in regard to photodetectors and photovoltaics are reviewed. Future research to modulate the Rashba strength is expected to promote the optoelectronic and spintronic applications of 2D perovskites.

## 3. Conclusions

In summary, this Special Issue mainly reports recent research progress regarding photofunctional nanomaterials and nanostructures that emit, manipulate, and harvest light. These contributions are expected to promote the development of integrated nanophotonic chips, hyperspectral imaging, photoelectrochemical water splitting, solar cells, and OLEDs. We hope that this Special Issue will help readers to gain more insight into this area and also provide helpful guidance for the future development of photofunctional nanomaterials and nanostructures. 
